# A new species of *Stigmatomma* from Taiwan (Hymenoptera, Formicidae, Amblyoponinae)

**DOI:** 10.3897/zookeys.705.10296

**Published:** 2017-10-03

**Authors:** Feng-Chuan Hsu, Flavia A. Esteves, Lien-Siang Chou, Chung-Chi Lin

**Affiliations:** 1 Institute of Ecology and Evolutionary Biology, National Taiwan University, No. 1, Sec. 4, Roosevelt Rd., Taipei 106, Taiwan; 2 California Academy of Sciences, 55 Music Concourse Drive, San Francisco, CA, U.S.A.; 3 Department of Biology, National Changhua University of Education, No. 1, Jin-De Rd., Changhua 500, Taiwan

**Keywords:** Cryptic species, dracula ants, Forest Dynamics Plot, Lienhuachih, soil sample, Winkler method

## Abstract

*Stigmatomma* is the most speciose ant genus in the subfamily Amblyoponinae. In the present paper, the worker caste of a new species is described, *S. luyiae* **sp. n.**, which was collected from a soil sample in a subtropical evergreen broad-leaved forest in Taiwan. An identification key to the females of *Stigmatomma* species with 11 antennomeres occurring in Asia is also provided.

## Introduction

*Stigmatomma* Roger (1859) is the most diverse ant genus in the subfamily Amblyoponinae (AntCat 2017); to date, 52 extant and two fossil species are considered valid. The genus was recently revived from a synonymy with the genus *Amblyopone* Erichson (1842) by Yoshimura and Fisher (2012), and is currently a senior synonym of *Arotropus* Provancher (1881) and of *Bannapone* Xu (2000) (Yoshimura and Fisher 2012, Ward and Fisher 2016).

*Stigmatomma* species are distributed in all bioregions except the neotropics. Previously, only four species had been recorded in Taiwan: *S. bruni* Forel (1912), *S. sakaii* (Terayama 1989), *S. silvestrii* Wheeler (1928), and *S. zaojun* (Terayama 2009). In Taiwan, as elsewhere, *Stigmatomma* species display a cryptic lifestyle, usually found in the leaf-litter, rotten wood, and in the soil of well-developed forests and forest edges (Terayama 2009). Given this, they are rarely collected.

In this paper, a new species for the genus *Stigmatomma* is described, *S. luyiae* sp. n., based on two workers collected from sifted soil, in Taiwan. In addition, an updated identification key to workers of the Asian *Stigmatomma* species with 11 antennomeres is presented.

## Materials and methods

### Images

A Leica DFC 425 camera mounted in a Leica Z16 APO and LEICA APPLICATION SUITE software (version 3.8; Leica Microsystems, Switzerland) were used for multifocus photography. HELICON FOCUS (version 6.6.1 Pro; Helicon Soft Ltd, Ukraine) rendered the extended focus montage images.

Electron micrographs of the uncoated holotype were obtained with a Hitachi SU3500 SEM (Hitachi High-Technologies, Japan) set to high vacuum mode (SEM mode), low accelerating voltage (1.5 kV), and spot intensity 40. Specimen preparation procedures were modified from Keller (2011): the point-mounted holotype was submerged in warm water to dissolve the mounting glue before being placed in 95% ethanol for two hours. It was then attached laterally to a SEM aluminum Zeiss stub (TED PELLA, INC., USA) via a double-sided adhesive conducting PELCO tab (TED PELLA, INC., USA), and left to air dry overnight before scanning.

All images were edited and enhanced on Adobe Photoshop CS6 (version 13.0.6 x64; Adobe Systems Incorporated, USA) and are available on AntWeb.org. Illustrations were created on Adobe Illustrator CS6 (version 16.0.4; Adobe Systems Incorporated, USA)

### Measurements and indices

Measurements were taken using a Leica M205C dissecting microscope (Leica Microsystems, Switzerland) with an ocular micrometer, and recorded to the nearest 0.01 mm. Abbreviations used in text are as follows:

**TL** Total Length: maximum length of the specimen in lateral view, measured from the anterior-most point of the mandibles to the apex of the abdominal segment VII, excluding the sting.

**HL** Head Length: length of the head in full-face view, excluding the mandibles; measured from the anterior clypeal margin to the midpoint of a transverse line connecting the posterior corners of the head.

**HW** Head Width: maximum width of the head in full-face view.

**HW2** Head width 2: in full-face view, width of the head immediately posterior to the posterolateral margin of the clypeus (as in Taylor 1978).

**SL** Scape Length: maximum length of the scape (basal-most antennomere), excluding the basal constriction and condyle.

**ML** Mandible length: outer length of the mandible (as in Taylor 1978).

**WL** Weber’s Length: diagonal length of the mesosoma in profile, measured from the base of the anterior slope of the pronotum to the metapleural lobe.

**PPW** Propodeal posterior width: width of the propodeum in dorsal view, measured across the posterior margin of the propodeum.

**PnW** Pronotal Width: maximum width of the pronotum in dorsal view.

**PtW** Petiole Width: maximum width of the petiolar tergite (abdominal tergite II) in dorsal view.

**PtL** Petiole Length: maximum diagonal length of the petiole in profile, measured from the anterior-most point of the subpetiolar process to the posterodorsal corner of the petiolar tergite.

**CI** Cephalic index = HW/HL × 100.

**MI** Mandibular index = ML/HL × 100.

**SI** Scape index = SL/HW × 100.

### Terminology

The terminology used follows prior studies: cuticular sculpture (Harris 1979), pilosity inclination (Wilson 1955), morphological terms (Keller 2011), and vestiture (Esteves and Fisher 2016).

### Depositories

**NMNS** National Museum of Natural Science, Taichung City, Taiwan.

**TARI** Taiwan Agricultural Research Institute, Taichung City, Taiwan.

## Taxonomy

### Stigmatomma luyiae sp. n.

Taxon classificationAnimaliaHymenopteraFormicidae

http://zoobank.org/AD739019-1E86-4A82-8516-229B4DECF8FD

#### Material.

Holotype: worker, pinned. Original label: “TAIWAN: Nantou County, LFDP, 10.i.2015, F. C. Hsu col. CASENT0922349” Deposited at NMNS.

Paratype: worker, pinned. Original label: “TAIWAN: Nantou County, LFDP, 10.i.2015, F. C. Hsu col. ANTWEB1032000” Deposited at TARI.

#### Type locality.

Lienhuachih Forest Dynamics Plot (LFDP), 23°55’01”N / 120°52’58”E, 770m, Nantou County, Taiwan, 10.i.2015, F. C. Hsu col., Winkler sample (sifted soil).

#### Diagnosis.

Workers of *Stigmatomma luyiae* can be distinguished from those of other *Stigmatomma* species by the combination of the following characters (asterisks flag putative unique characters within *Stigmatomma*):

1. Eleven antennomeres.

2. Mandibles as long as the head (MI: 100).

3. Mandibles’ baso-masticatory margin with a single row of teeth.

4. Mandibles with longer, jigsaw-tab-shaped median teeth.

5. *Anterior clypeal margin flat; modified setae or tubercular cuticular projections absent.

6. Anterior portion of the median area of the clypeus with seven stout, acuminate flattened-apex setae arranged in a single transversal row.

7. Frontal lobes closely approximated; median area of the clypeus extending posteriorly as a narrow longitudinal strip between the antennal sockets.

8. Antennal scrobe absent.

9. Genal teeth present, but minute.

10. Dorsal face of the head densely costate-foveolate, slightly catenated.

11. Head quadrate (CI: 100).

12. Mesepisternum divided into anepisternum and katepisternum.

13. Lamella absent on the ventral margin of the calcar of strigil.

14. *Anterior face of mesobasitarsus with round sulcus filled with microvilli-like projections.

15. One metatibial spur.

16. Sulcus absent on the anterior face of the metabasitarsus.

17. Fenestra absent on the subpetiolar process.

18. Stout spiniform setae absent on the hypopygium.

#### Description.

**Figure 1.**
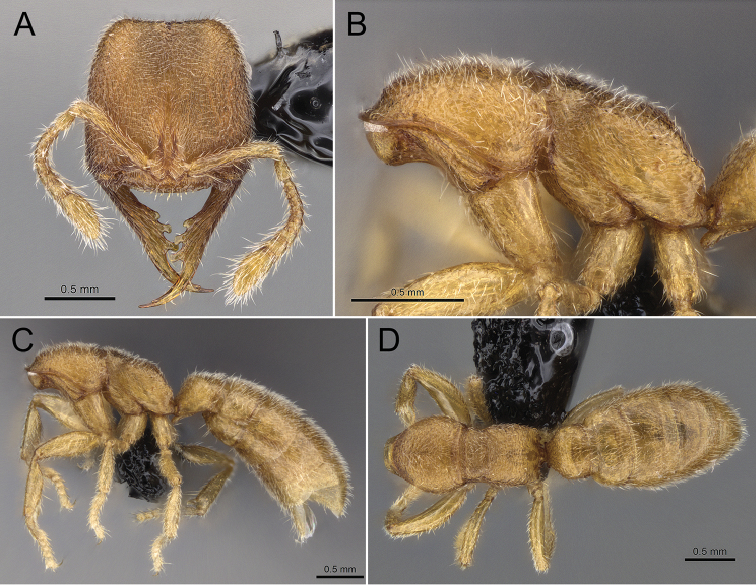
Holotype of *Stigmatomma luyiae* sp. n. (CASENT0922349); worker. Images by FAE; available at AntWeb.org. **A** Full-face view **B** Mesosoma, lateral view **C** Lateral view **D** Dorsal view.

Holotype measurements (Figure 1): TL: 2.45 mm, HL: 0.5 mm, HW: 0.5 mm, HW2: 0.47 mm; SL: 0.3 mm, ML: 0.5 mm, WL: 0.6 mm, PPW: 0.24 mm, PnW: 0.31 mm, PtW: 0.24 mm, PtL: 0.16 mm, CI: 100, MI: 100, and SI: 60.

Paratype measurements: TL: 2.43 mm, HL: 0.48 mm, HW: 0.48 mm, HW2: 0.45 mm, SL: 0.29 mm, ML: 0.48 mm, WL: 0.6 mm, PPW: 0.22 mm, PnW: 0.32 mm, PtW: 0.24 mm, PtL: 0.16 mm, CI: 100, MI: 100, and SI: 60.

**Figure 2.**
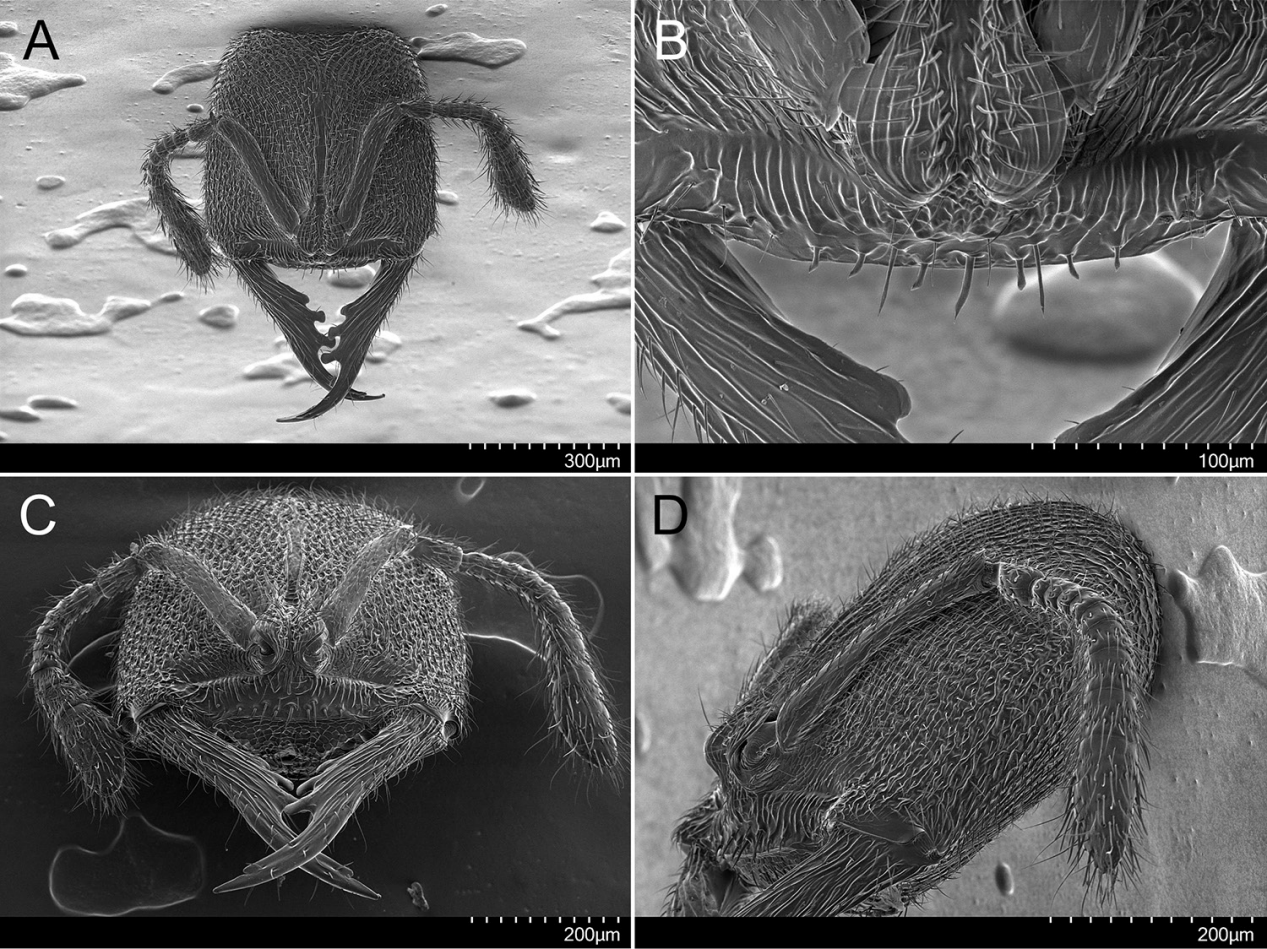
Holotype of *Stigmatomma luyiae* sp. n. (CASENT0922349); worker. Images by FAE; available at AntWeb.org. **A** Full-face view **B** Anterior margin of clypeus, dorsal view **C** Mandibles, anterior view **D** Head, lateral view.

***Head.*** Dorsal face of the head quadrate (CI: 100), bearing longer erect to suberect hairs, and numerous shorter suberect to subdecumbent pilosity; sculpture densely costate-foveolate, slightly catenated (Figs 1A, 2A). Posterior margin of the head slightly concave in full-face view. Antenna with eleven antennomeres, bearing abundant erect to subdecumbent pilosity (Figs 1A, 2C–D). Frontal lobes closely approximated: median area of the clypeus extending posteriorly as a narrow longitudinal strip between the antennal sockets (Figs 1A, 2B–C). Antenna with eleven antennomeres (Figs 1A, 2A, C–D). Antennal scrobes absent (Figs 1A, 2A, C). Compound eyes absent (Figs 2C, D). Genal teeth present, but minute. Anterior clypeal margin flat; modified setae or tubercular cuticular projections absent (Figure 2B). Anterior portion of the median area of the clypeus with seven stout, acuminate flattened-apex setae arranged in a single transversal row; longer median seta; each seta rises from a minute tubercle-like cuticular projection (Figure 2B). Mandibles elongated, falciform; baso-masticatory margin with a single row of five teeth (Figs 1A, 2A). Mandibular teeth arrangement, from base to apex: smaller blunt tooth; two longer jigsaw-tab-shaped teeth; long diastema; indistinct, acute pre-apical tooth, immediately followed by an acute apical tooth (Figs 1A, 2A, C). Dorsal face of the mandibles costate (Figs 1A, 2A). Mandibles with erect to subdecumbent pilosity (Figs 1A, 2A). Mandibles as long as the head (MI: 100).

**Figure 3.**
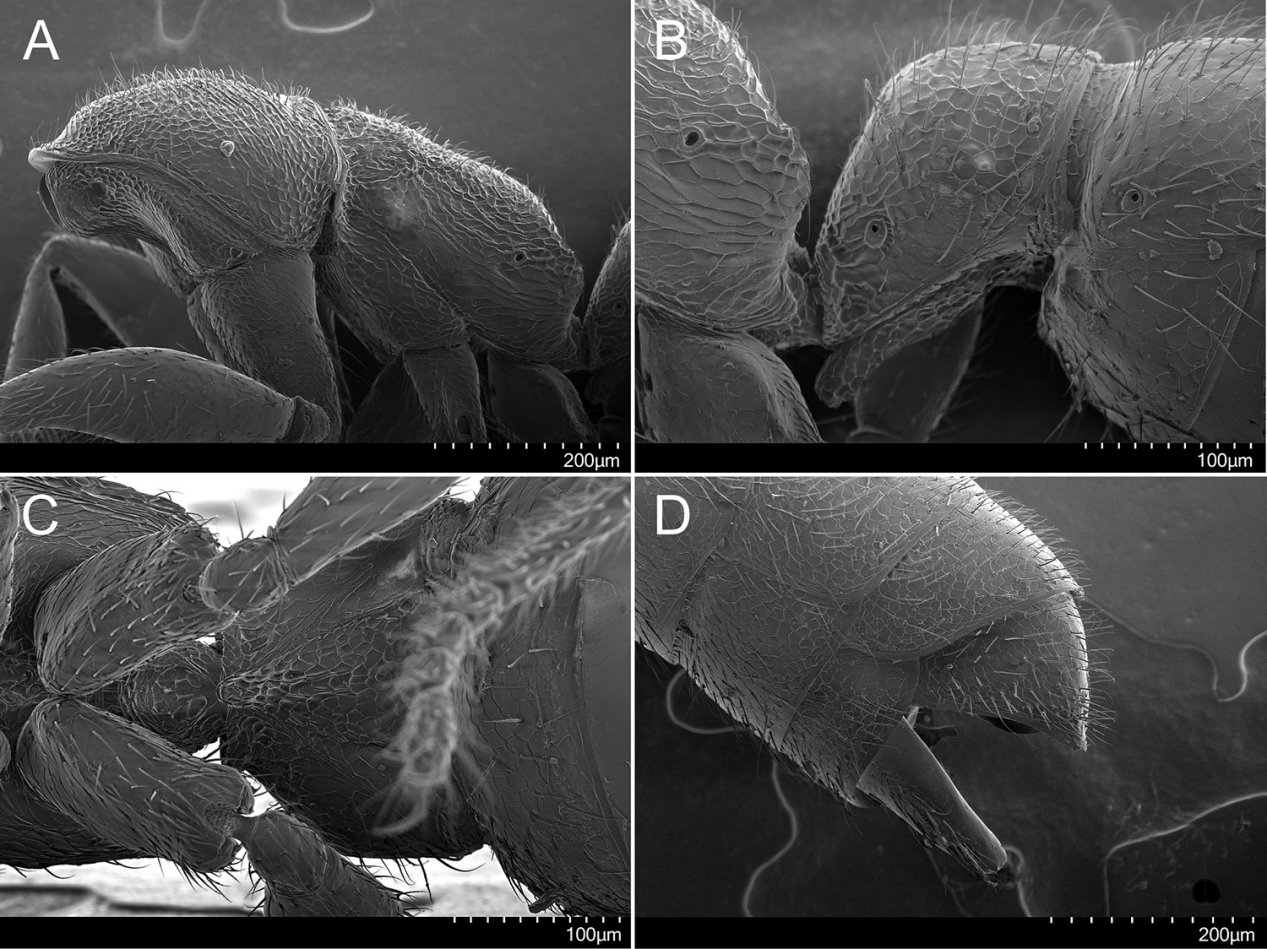
Holotype of *Stigmatomma luyiae* sp. n. (CASENT0922349); worker. Images by FAE; available at AntWeb.org. **A** Mesosoma, lateral view **B** Petiole, lateral view **C** Petiole, ventral view **D** Apex of the gaster, lateral view.

***Mesosoma.*** Mesepisternum divided into anepisternum and katepisternum (Figs 1B, 3A). Metanotal suture present (Figure 1D). In profile, lateral margins of propodeal declivitous face are not continuous: ventral portion is raised (Figs 1B, 3A). In the dorsal view, pronotum, mesonotum, and propodeum strigate-rugulose; declivitous face of the propodeum weakly strigate-rugulose. Posterolateral portion of the pronotum, mesepisternum, and posterolateral face of propodeum areolate; remainder of the lateral face of the propodeum strigate-rugulose-somewhat areolate; metapleuron imbricate (Figure 3A). Mesosoma covered with erect to subdecumbent pilosity (Figure 3A).

**Figure 4.**
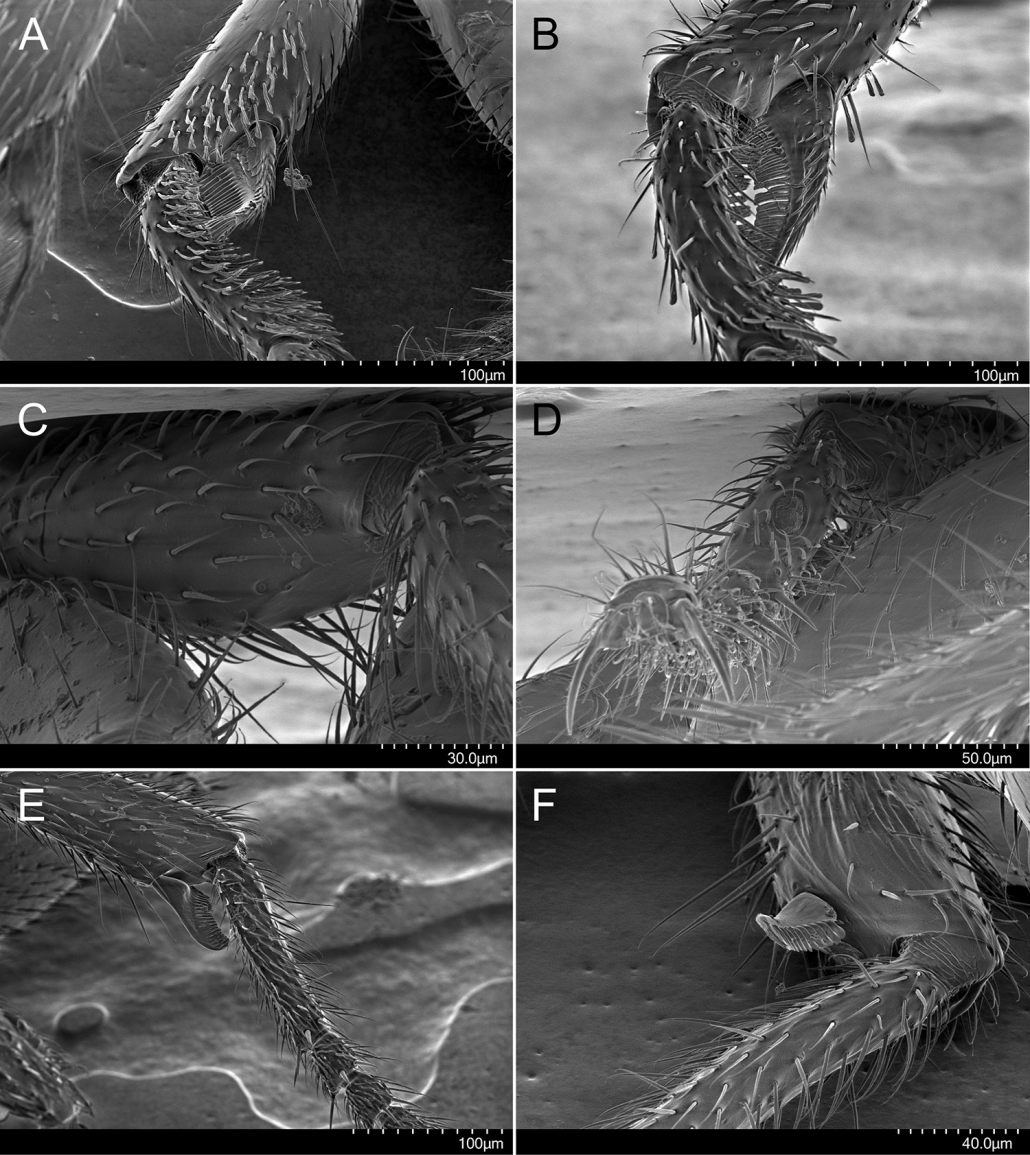
Holotype of *Stigmatomma luyiae* sp. n. (CASENT0922349); worker. Images by FAE; available at AntWeb.org. **A** Foreleg, anterior face. Close-up of the protibial apex, including the calcar of strigil, and probasitarsus **B** Foreleg, posterior face. Close-up of the protibial apex, including the calcar of strigil, and probasitarsus **C** Midleg, ventral face. Close-up of the mesotibial apex **D** Midleg, anterior face. Close-up of the mesobasitarsus, which bears a round sulcus filled with microvilli-like projections **E** Hindleg, anterior face. Close-up of the metatibial apex, including the metatibial spur, and metabasitarsus **F** Hindleg, posterior face. Close-up of the metatibial apex, including the metatibial spur, and basal portion of metabasitarsus.

***Legs.*** Lamella absent on the ventral margin of the calcar of strigil (Fig. 4A, B). Anterior face of calcar of strigil with squamiform microtrichia; posterior face with lanceolate microtrichia (Fig. 4A–B). Multiple spatulate, carinate setae on the anterior face of protibial apex, next to calcar of strigil (Figure 4A). Multiple spatulate, carinate setae on the anterior face of probasitarsus (Figure 4A). Mesotibial spur absent (Figure 4C). Anterior face of mesobasitarsus with round sulcus filled with microvilli-like projections (Figure 4D). One metatibial spur; pectinate; anterior and posterior faces glabrous (Fig. 4E–F). Sulcus absent on the anterior face of the metabasitarsus (Figure 4E). Arolium present on pro-, meso-, and metapretarsus.

***Metasoma.*** Subpetiolar process well developed and lobe-shaped; fenestra absent (Figs 1C, 3B). Prora present (Figs 1C, 3B). Stout spiniform setae absent on hypopygium (Figure 3D). Petiolar tergite, laterotergite, and poststernite areolate/imbricate (Figs 3B–C). Gaster imbricate; mostly covered with suberect pilosity (Figs 1C, 3B, D).

***Color.*** Head color orange-brown; body yellow-brown; apex of gaster and appendages yellowish (Figure 1).

#### Etymology.

The name *luyiae* is homage to Miss Lu-Yi Wang. The fieldwork that yielded specimens for this study could not have been completed without her participation.

#### Other castes.

Unknown.

#### Distribution.

**Figure 5.**
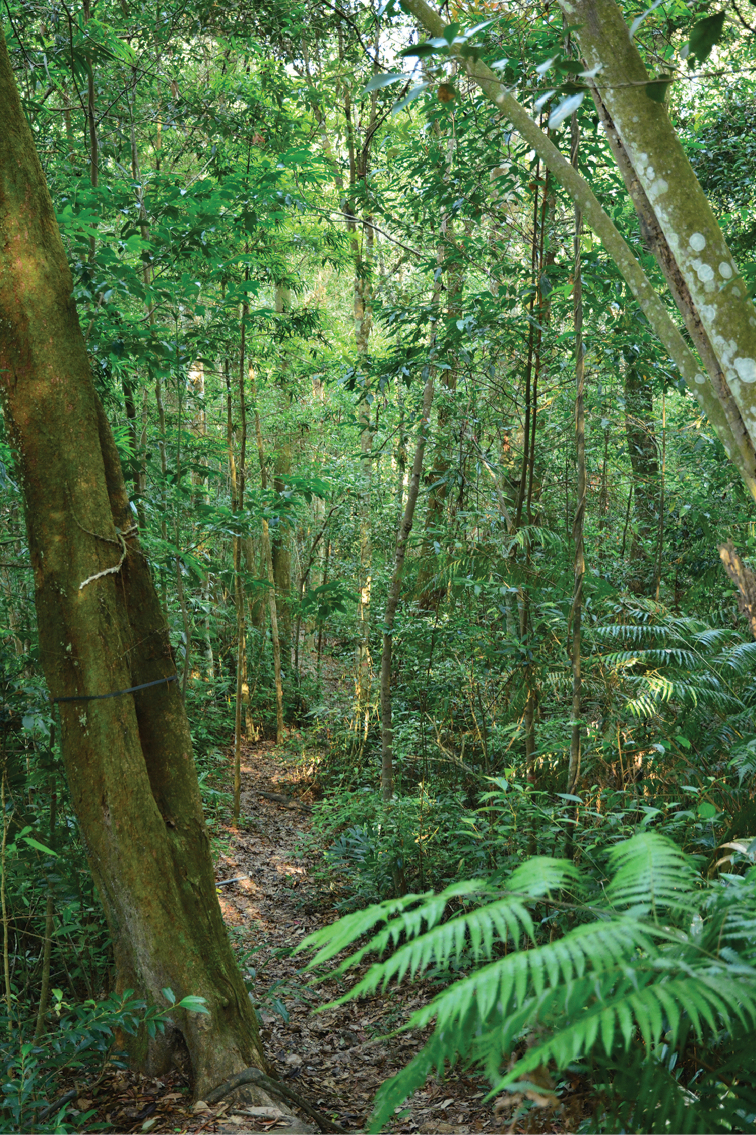
Photograph of the subtropical evergreen broad-leaved forest in the Lienhuachih Forest Dynamics Plot, Nantou County, Taiwan.

To date, *Stigmatomma luyiae* sp. n. was only collected in the soil of a subtropical evergreen broad-leaved forest, which is part of the *Machilus-Castanopsis* vegetation zone, in Taiwan (Figure 5).

#### Discussion.

*Stigmatomma luyiae* is easily distinguished from most of its congeners in Asia due to its mandibles, which present only a single row of teeth instead of two rows. The only *Stigmatomma* species in that region sharing this character with the new species are those formerly classified as *Bannapone* (Eguchi et al., 2015): *S. caliginosum* (Onoyama, 1999), *S. crypticum* (Eguchi et al., 2015), *S. fulvidum* (Terayama, 1987), *S. mulanae* (Xu, 2000), *S. pertinax* (Baroni Urbani, 1978), and *S. scrobiceps* (Guenard et al., 2013). Within this group, *S. fulvidum* possesses antennae with twelve antennomeres (Terayama 1987), while the antennae of the other species, including *S. luyiae*, have eleven antennomeres. Only *S. luyiae* and *S. mulanae* possess the median mandibular teeth in a jigsaw tab-shaped arrangement (Fig. 8A–B).

The diagnostic characters for setting apart *Stigmatomma luyiae* from *S. mulanae* are the presence/absence of cuticular projections and modified setae on the anterior margin of the clypeus, head shape, and relative size of the mandibles. In *S. luyiae*, the anterior clypeal margin is flat and bears neither tubercular projections nor stout setae. Instead, stout, acuminate flattened-apex setae rise from the anterior portion of the median area of the clypeus (Figure 2B). Also, the head is quadrate (CI: 100), and the mandibles are as long as the head (MI: 100). In *S. mulanae*, the anterior margin of the clypeus presents tubercle-like cuticular projections, and each of which bears a stout, conic seta (Figure 8A); the head is rectangular (CI: 84; Xu 2000); and the mandibles are shorter than the head (ML: 0.30, HL: 0.38; Xu 2000).

### List of known *Stigmatomma* species in Taiwan

*Stigmatomma bruni* Forel

*Stigmatomma luyiae* sp. n.

*Stigmatomma sakaii* (Terayama)

*Stigmatomma silvestrii* Wheeler

*Stigmatomma zaojun* (Terayama)

### List of known species of Asian *Stigmatomma* species with 11 antennomeres

*Stigmatomma caliginosum* (Onoyama)

*Stigmatomma crypticum* (Eguchi, Bui, Yamane & Terayama)

*Stigmatomma luyiae* sp. n.

*Stigmatomma mulanae* (Xu)

*Stigmatomma pertinax* (Baroni Urbani)

*Stigmatomma sakaii* (Terayama)

*Stigmatomma scrobiceps* (Guénard, Blanchard, Liu, Yang & Economo)

*Stigmatomma xui* Bharti & Rilta

### Identification key to the females of the Asian *Stigmatomma* species with 11 antennomeres

This key is modified from the identification keys provided by Bharti and Rilta (2015), Eguchi et al. (2015), and Xu and Chu (2012).

**Table:** 

1	Baso-masticatory margin of mandibles with two parallel rows of teeth, or with bifid teeth arranged in a single row	**2**
–	Baso-masticatory margin of mandibles with undivided teeth arranged in a single row	**3**
2.	In full-face view, head trapezoidal: anterior margin wider than posterior margin. Baso-masticatory margin of mandible with five sets of paired teeth (Figure 6A)	***S* . *sakaii***
–	In full-face view, head rectangular: anterior and posterior margins with same width. Baso-masticatory margin of mandible with four sets of paired teeth (Figure 6B)	***S. xui***
3	Frontal carinae long, surpassing mid-length of the head. Antennal scrobe present	**4**
–	Frontal carinae short, not surpassing mid-length of the head. Antennal scrobe absent	**5**
4	Mandible bearing four teeth (including indistinct pre-apical tooth, and apical tooth). Anterior clypeal margin bearing five stout, dentiform setae. Frontal lobes relatively separated by median portion of clypeus (Figure 7A)	***S* . *scrobiceps***
–	Mandible bearing six teeth (including pre-apical and apical teeth). Anterior clypeal margin bearing seven stout, dentiform setae. Frontal lobes closely approximated (Figure 7B)	***S* . *crypticum***
5	Median mandibular teeth jigsaw-tab-shaped	**6**
–	Median mandibular shaped otherwise, but never as jigsaw-tabs	**7**
6	Clypeal setae rising from small tubercular projections of the anterior clypeal margin. Head rectangular, longer than wide (CI: 84; Xu 2000). Mandibles shorter than head (ML: 0.30, HL: 0.38; Xu 2000) (Figure 8A)	***S. mulanae***
–	Clypeal anterior margin flat: clypeal setae rise from the anterior portion of the median area of the clypeus, not from the clypeal anterior margin. Head quadrate, as long as wide (CI: 100). Mandibles as long as head (MI: 100) (Figure 8B)	***S. luyiae* sp. n.**
7	In full-face view, head trapezoidal: anterior margin much wider than posterior margin; slightly broader than long (CI: 101.9; Baroni Urbani 1978). Posterior margin of the head concave. Mandibular pre-apical tooth comparatively large. Anterior clypeal margin bearing eight stout, dentiform setae (Figure 9A)	***S* . *pertinax***
	In full-face view, head rectangular: anterior margin as wide as posterior margin; longer than broad (CI: 82-88; Onoyama 1999). Posterior margin of the head straight. Mandibular pre-apical tooth indistinct. Anterior clypeal margin bearing five stout, dentiform setae (Figure 9B)	***S* . *caliginosum***

**Figure 6.**
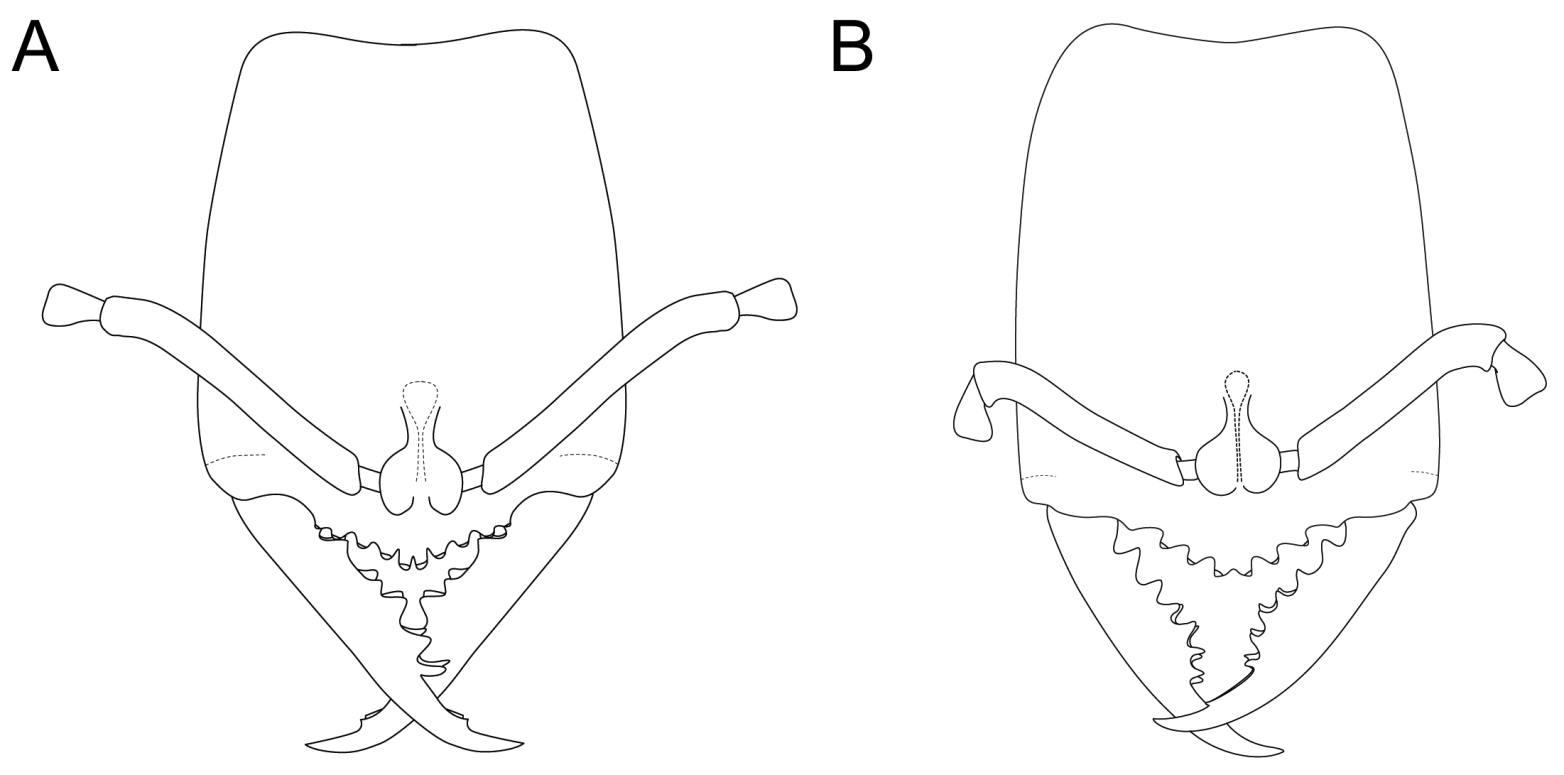
*Stigmatomma sakaii* and *S. xui*. Illustrations by FAE. **A** *S. sakaii*; worker; full-face view **B** *S. xui*; worker; full-face view.

**Figure 7.**
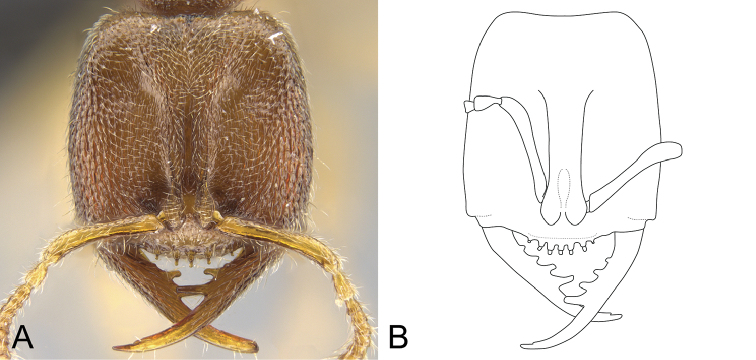
*Stigmatomma scrobiceps* and *S. crypticum*. **A** Holotype of *S. scrobiceps* (CASENT0339957); worker; full-face view. Image by Michele Esposito; available at AntWeb.org **B** *Stigmatomma crypticum*; worker; full-face view. Illustration by FAE.

**Figure 8.**
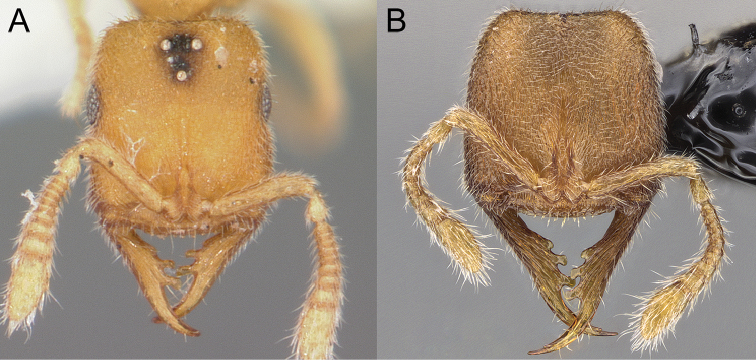
*Stigmatomma mulanae* and *S. luyiae* sp. n. **A** Holotype of *S. mulanae* (CASENT0104980); queen; full-face view. Image by April Nobile; available at AntWeb.org **B** Holotype of *S. luyiae* (CASENT0922349); worker; full-face view. Image by FAE; available at AntWeb.org.

**Figure 9.**
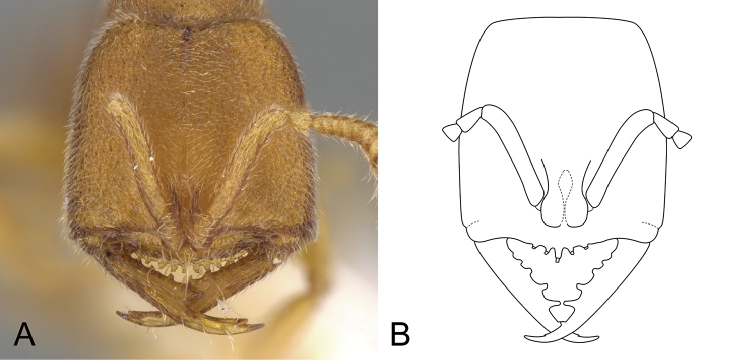
*Stigmatomma pertinax* and *S. caliginosum*. **A** Holotype of *S. pertinax* (CASENT0906831); worker; full-face view. Image by Michele Esposito; available at AntWeb.org **B** *S. caliginosum*; worker; full-face view. Illustration by FAE.

## Supplementary Material

XML Treatment for Stigmatomma luyiae
